# LncRNA PCNAP1 Promotes Hepatoma Cell Proliferation through Targeting miR-340-5p and is Associated with Patient Survival

**DOI:** 10.1155/2021/6627173

**Published:** 2021-04-28

**Authors:** Miao He, Lingjing Hu, Ping Bai, Tingting Guo, Nan Liu, Fei Feng, Jin Zhang

**Affiliations:** ^1^Department of Oncology and Hematology, Chongqing Traditional Chinese Medicine Hospital, Chongqing 400011, China; ^2^Department of Critical Care Medicine, Chongqing Traditional Chinese Medicine Hospital, Chongqing 400011, China

## Abstract

Hepatocellular carcinoma (HCC) is one of the most common malignancies and causes poor outcome. Dysregulation of long noncoding RNA (lncRNA) is involved in HCC. Upregulation of the lncRNA PCNAP1 has been reported to promote HBV-infectious HCC growth, but its clinical significance and underlying mechanisms in HCC development remain unclear. Here, we report that PCNAP1 expression is increased in both HBV-infectious and noninfectious HCC tissues compared with matched normal tissues, and its upregulation correlates with poor survival rates of HCC patients. Furthermore, we found that PCNAP1 promotes HCC cell proliferation through acting as a competitive endogenous RNA (ceRNA) to sponge miR-340-5p, which has been reported to directly inhibit ATF7 expression in HCC cells. Moreover, the PCNAP1/miR-340-5p/ATF7 signaling associates with the poor survival rates of HCC patients. Collectively, our findings suggest that the PCNAP1/miR-340-5p/ATF7 signaling may be a potential biomarker for the prognosis of HCC patients and a potential therapeutic target for HCC.

## 1. Introduction

Liver cancer is the fifth leading cause of cancer-related deaths in the United States in 2019, with 42,030 estimated new cases and 31,780 estimated deaths [1]. Hepatocellular carcinoma (HCC) accounts for 90% of primary liver cancers, which result from chronic infection with hepatitis B virus (HBV), alcohol abuse, and so forth [2]. Approximately, 40–50% of patients with early HCC can be cured, but for advanced-stage HCC, patients hardly benefit from therapy [3, 4]. Sorafenib, a multitarget tyrosine kinase inhibitor (TKI), has antiangiogenic and antiproliferative effects and can extend the overall survival of patients with advanced-stage HCC from 8 to 11 months [5], but HCC becomes resistant to sorafenib after that time [6]. Therefore, it is necessary to explore novel diagnostic biomarkers and therapeutic targets for HCC.

LncRNAs are a class of single RNAs with more than 200 nucleotides in length and cannot encode proteins. Via exploration for a decade, lncRNAs have been shown to be involved in many kinds of human diseases, including almost all types of cancers. LncRNAs participate in regulating multiple phenotypes of tumor cells, such as cell proliferation [7], migration [8], invasion [9], adhesion [10], and stem cell-like properties [11]. Increasing numbers of lncRNAs have been reported to be involved in HCC. For example, lncTCF7 promotes self-renewal of human liver cancer stem cells [12]; lncRNA-ATB promotes the invasion metastasis cascade in HCC [13]. LncRNA PSTAR inhibits HCC cell proliferation and tumorigenicity via inducing p53-mediated cell cycle arrest [14]. Recently, Feng et al. reported that lncRNA PCNAP1 is associated with HBV-infectious HCC patients and acts as a competitive endogenous RNA (ceRNA) to sponge miR-154, therefore regulating the expression of PCNA, which interacts with HBV cccDNA, finally, enhancing HCC growth. However, the clinical significance of PCNA and its regulatory mechanism except for HBV remain unclear.

In the present study, we found that PCNAP1 expression is increased in HCC tissues and HBV-infectious HCC tissues compared with matched normal tissues and is correlated with the overall survival of patients with HCC. Furthermore, we found that PCNAP1 promotes HCC cell proliferation via miR-340-5p/ATF7 signaling. Our findings suggest that PCNAP1 may be a prognostic biomarker and therapeutic target for HCC.

## 2. Materials and Methods

### 2.1. Patients and Specimen

A total of 92 patients with HCC were enrolled in this study, including 41 HCC patients without infection and 51 ones with HBV infection at Chongqing Traditional Chinese Medicine Hospital (Chongqing, China) from 2013 to 2015 (Table 1). HCC tissues and matched normal tissues were collected in the operation room and stored in liquid nitrogen within 15 min. The matched normal tissues were collected 2 cm away from the tumor. All tissues were examined histologically. All patients did not undergo therapeutics, including chemotherapy and radiotherapy, before operation. The follow-up of 92 patients was carried out.

### 2.2. Cell Culture

Hepatoma cell line, SNU-182, was purchased from American Type Culture Collection (ATCC, Manassas, Virginia, USA). Cell was cultured in RPMI-1640 Medium (HyClone, Logan, Utah, USA) supplemented with 10% Fetal Bovine Serum (FBS, HyClone) at 37°C in 5% CO_2_.

### 2.3. RNA Isolation and Quantitative Reverse Transcription Polymerase Chain Reaction (qRT-PCR)

Total RNAs were extracted from cells or tissues using Trizol reagent (Invitrogen, Carlsbad, CA, USA). Reverse transcription assays were performed using a PrimeScript™ RT reagent kit (TaKaRa, Japan) and quantitative PCR was performed using SYBR® Premix Ex Taq™ II (TaKaRa) according to the manufacturer's instructions. GAPDH serves as an internal control for PCNAP1 and U6 for miR-340-5p. The expression data were analyzed using the 2^−ΔΔCT^ method. All experiments were carried out in duplicate and repeated twice. The primer sequences are shown in Table 2.

### 2.4. Cell Proliferation

SNU-182 cells were seeded in 96-well plates, 2 × 10^3^ cells per well. Cell proliferation was detected once a day for five days using Cell Counting kit 8 (CCK8, Beyotime, Shanghai, China) according to the manufacturers' instructions.

### 2.5. Oligonucleotide Transfection

Oligonucleotides, including PCNAP1 siRNA, PCNAP1-overexpressing vector, miR-340-5p mimics, and miR-340-5p inhibitors, were transfected into cells using Lipofectamine 3000 reagent (Thermo Fisher Scientific, Shanghai, China) according to the manufacturer's protocol. Twenty-four hours after transfection, cells were isolated for the next experiments. The oligonucleotide sequences are shown in Table 2.

### 2.6. Western Blotting

Total proteins were extracted from cells and tissues using RIPA Lysis and Extraction Buffer (Thermo Fisher Scientific). Proteins were separated via 12% SDS-PAGE gels and then transferred onto PVDF (Polyvinylidene Fluoride) membrane (Darmstadt, Germany), followed by being blocked by three percent Bovine Serum Albumin (BSA, Thermo Fisher Scientific) for 1 hour. The PVDF membranes were incubated with anti-ATF7 primary antibody (1 : 1000) (Abcam Biotechnology (ab183507), UK) or anti-GAPDH (1 : 5000) (Abcam Biotechnology (ab8245)) overnight at 4°C. Then, goat anti-rabbit or mouse secondary antibodies (1 : 10000) (Santa Cruz Biotechnology, USA) were used for 1 hour at room temperature, respectively. GAPDH was used to normalize protein expression.

### 2.7. Vector Construct

PCNAP1 fragment containing wild type or mutant of the binding sites of miR-340-5p was synthesized with flanking *Spe I* and *Hind III* restriction enzyme digestion sites, respectively, and then inserted into pMIR-reported luciferase vectors. PCNAP1 full-length sequence was synthesized with flanking *Spe I* and *Hind III* restriction enzyme digestion sites, followed by insertion into the pcDNA3.1 vector.

### 2.8. Luciferase Report Assay

HEK293 T cells were cultured in 12-well plates to reach a 70% confluent rate and then transfected with Oligonucleotides, including the constructed vectors miR-340-5p mimic and/or renilla luciferase control vector (pRL-TK, Promega, Madison, WI, USA), as well as the corresponding controls, using Lipofectamine 3000 (Thermo Fisher Scientific). Twenty-four hours after transfection, fluorescence activity was analyzed via GloMax 20/20 Luminometer. Renilla luciferase activity serves as an internal control.

### 2.9. Statistical Analysis

All data are presented as mean ± standard deviation (SD). Comparison between two groups was analyzed via the Mann–Whitney–Wilcoxon test or two-tailed Student's *t*-test. The Kaplan–Meier method was used to analyze patient overall survival. Spearman's correlation test was used to analyze the correlation of miR-340-5p expression with PCNAP1 expression. *P* < 0.05 was considered statistically significant. All statistical analyses were performed using SPSS 19.0 (Chicago, IL, USA) and GraphPad Prism 8.0 (Graphpad Software Inc, California).

## 3. Results

### 3.1. PCNAP1 Expression Is Frequently Increased in Noninfectious and HBV-Infectious HCC Tissues

**Figure 1:**
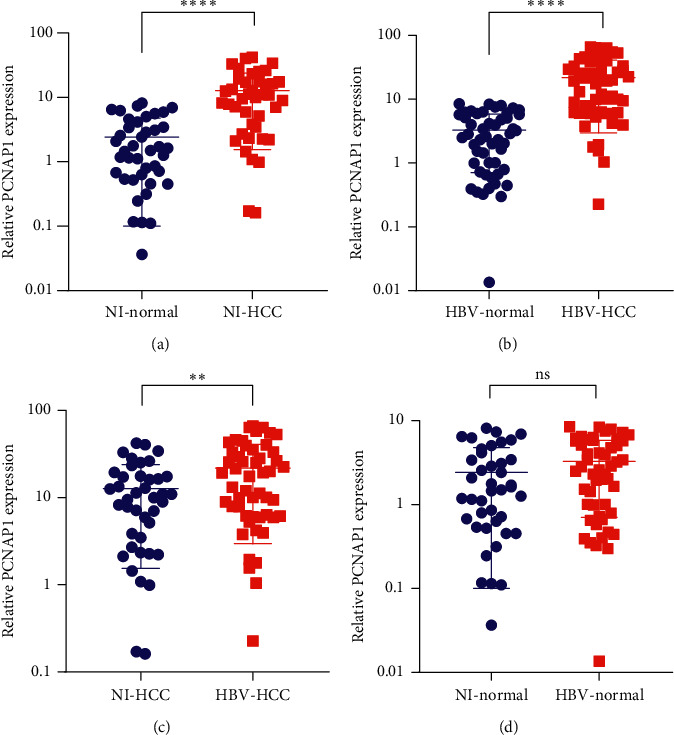
PCNAP1 expression is frequently increased in noninfectious and HBV-infectious HCC tissues. (a) Scatter plots show relative PCNAP1 expression in 41 pairs of noninfectious (NI) HCC tissues and the matched normal tissues. (b) Scatter plots show relative PCNAP1 expression in 51 pairs of HBV-infectious HCC tissues and the matched normal tissues. (c) Scatter plots show relative PCNAP1 expression in 41 noninfectious HCC tissues and 51 HBV-infectious HCC tissues. (d) Scatter plots show relative PCNAP1 expression in 41 noninfectious normal tissues and 51 HBV-infectious normal tissues. QRT-PCR was used to detect the expression of PCNAP1, and GAPDH served as the internal control. “^∗∗∗∗^”, *P* < 0.0001; “^∗∗^”, *P* < 0.01.

To investigate PCNAP1 expression in noninfectious and HBV-infectious HCC tissues, we collected 92 pairs of samples, including 41 noninfectious and 51 HBV-infectious HCC tissues and their matched normal tissues, from 92 patients with HCC. QRT-PCR experiments showed that PCNAP1 expression was significantly increased in noninfectious HCC tissues versus matched normal tissue (Figure 1(a)) and was significantly increased in HBV-infectious HCC tissues versus matched normal tissue (Figure 1(b)). Moreover, PCNAP1 expression was significantly increased in HBV-infectious HCC tissues versus noninfectious HCC tissues (Figure 1(c)). However, PCNAP1 expression was not significantly different between noninfectious normal tissue and HBV-infectious normal tissue (Figure 1(d)). These suggest that PCNAP1 expression is increased in noninfectious and HBV-infectious HCC tissues in turn, compared with the matched normal tissue, and PCNAP1 may be associated with HBV infection in HCC.

### 3.2. PCNAP1 Expression Signature is Correlated with Overall Survival of Patients with HCC

**Figure 2:**
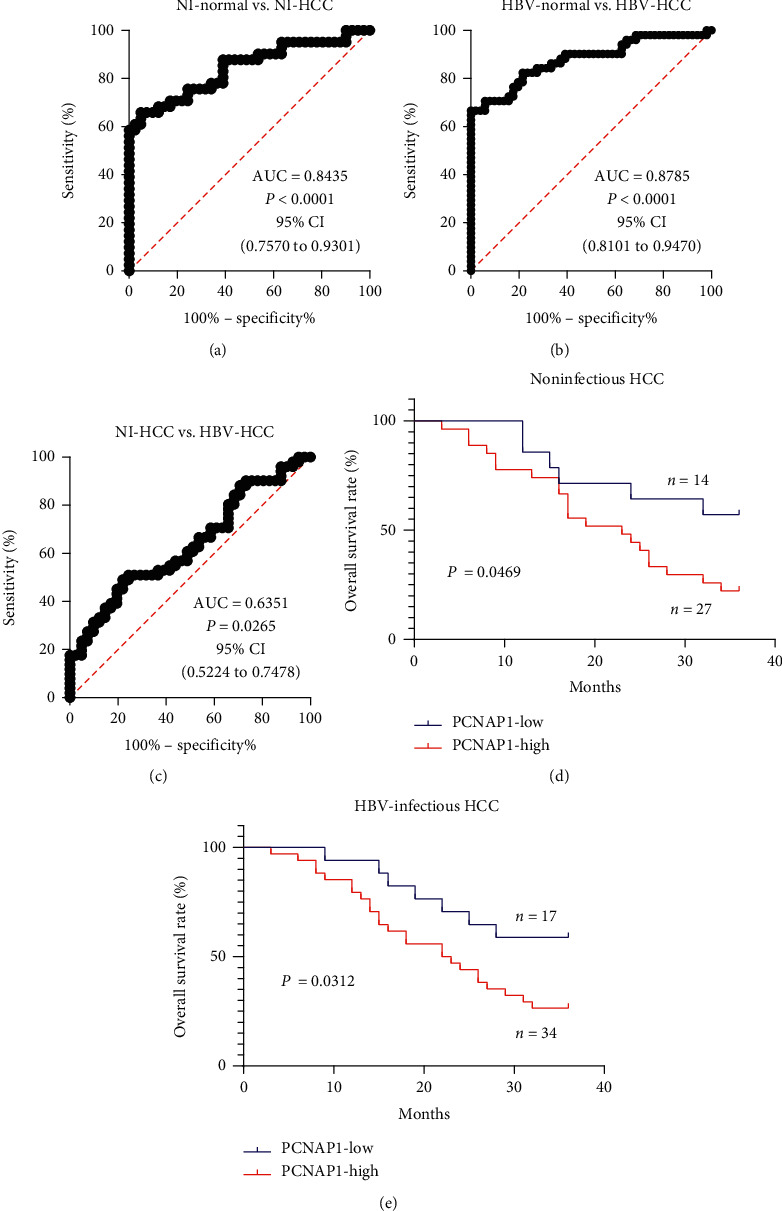
PCNAP1 expression signature is correlated with the overall survival of patients with HCC. ROC analyses show the values of AUC, sensitivity, and specificity in three groups of samples: (a) 41 noninfectious normal tissues versus 41 noninfectious HCC tissues; (b) 51 HBV-infectious normal tissues versus 51 HBV-infectious HCC tissues; (c) 41 noninfectious HCC tissues versus 51 HBV-infectious HCC tissues. (d) Survival analysis reveals that 27 noninfectious HCC patients with high expression of PCNAP1 had poorer overall survival rates than 14 those with low expression. (e) Survival analysis also shows that 34 HBV-infectious HCC patients with high expression of PCNAP1 had poorer overall survival rates than those 17 with low expression.

To investigate the clinical significance of PCNAP1 in HCC patients, we analyzed the correlation of PCNAP1 expression with the overall survival of HCC patients. ROC analyses revealed that PCNAP1 expression signature has an AUC of 0.8435 in distinguishing noninfectious HCC tissue from the matched normal tissue, with 65.85% sensitivity and 95.12% specificity and (1) a cut-off value of 6.985 (Figure 2(a)), an AUC of 0.8785 in HBV-infectious HCC tissue versus the matched normal tissue, with 66.67% sensitivity and 100% specificity; (2) a cut-off value of 8.725 (Figure 2(b)), an AUC of 0.6351 in HBV-infectious HCC tissue versus noninfectious HCC tissue, with 49.02% sensitivity and 78.05% specificity; and (3) a cut-off value of 18.42 (Figure 2(c)). Based on the cut-off values, the high and low expression of PCNAP1 were defined, respectively. Survival analyses showed that noninfectious HCC patients with high expression of PCNAP1 had poorer overall survival rates than those with low expression (Figure 2(d)), while HBV-infectious HCC patients with high expression of PCNAP1 also had poorer overall survival rates than those with low expression (Figure 2(e)). However, if the (3) cut-off value is used, the PCNAP1 expression signature will not distinguish the overall survival of either HBV-infectious or noninfectious HCC patients (data not shown). These suggest that PCNAP1 expression signature may predict the prognosis of HCC patients with either noninfection or HBV infection.

### 3.3. PCNAP1 Promotes HCC Cell Proliferation *In Vitro*

**Figure 3:**
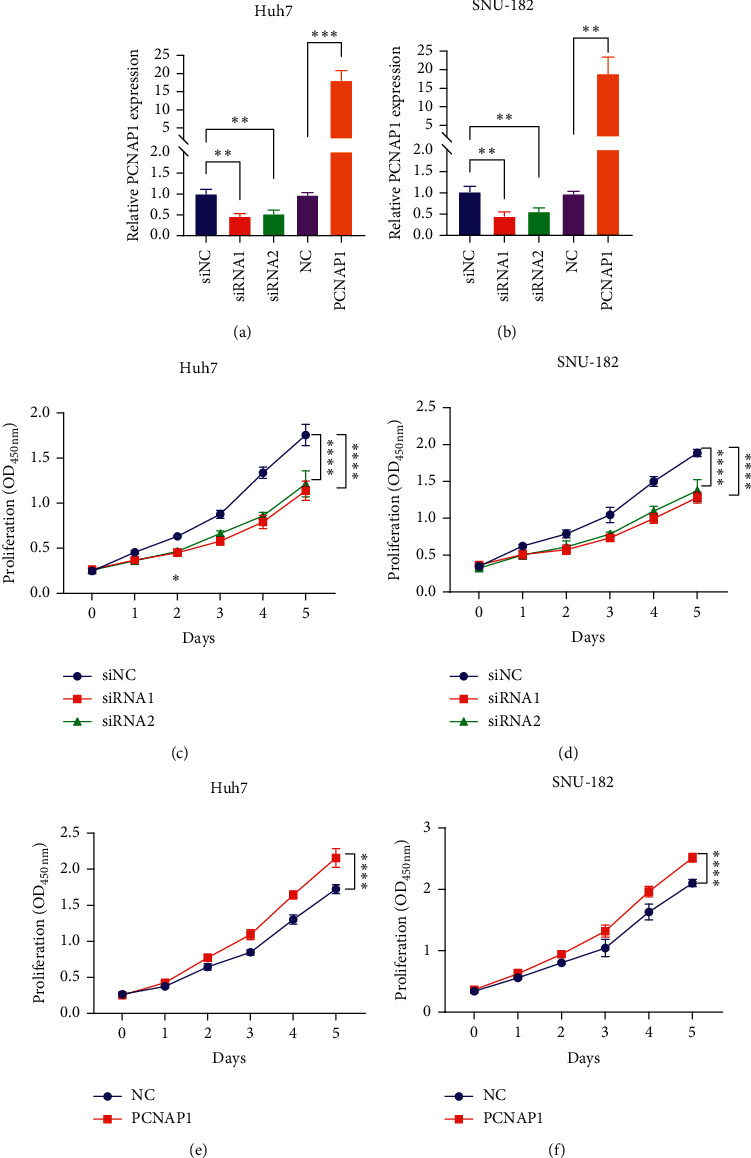
PCNAP1 promotes HCC cell proliferation *in vitro.* (a and b) Bars show the relative expression of PCNAP1 in Huh7 and SNU-182 cells which had been transfected with siNC, siRNA1, siRNA2, NC, or PCNAP1-overexpressing vectors, respectively. QRT-PCR was used to detect PCNAP1 expression, and GAPDH served as the internal control. (c–f) 24 h after transfection, the proliferation of Huh7 and SNU-182 cells at the zeroth to fifth day was measured using a CCK8 reagent. “^∗∗^”, *P* < 0.01; “^∗∗∗^”, *P* < 0.0001; “^∗∗∗∗^”, *P* < 0.0001.

A previous study has indicated that PCNAP1 promotes the proliferation of hepatoma cell lines, HepG2 and HepG2.2.15 [15]. To investigate whether PCNAP1 also influences the proliferation of other hepatoma cell lines, we selected two hepatoma cell lines, Huh7 and SNU-182. We performed gain- or loss-of-function PCNAP1 in the two cell lines using the PCNAP1-overexpressing vector or specific siRNAs, respectively (Figures 3(a) and 3(b)). CCK8 experiments showed that PCNAP1 knockdown significantly inhibited Huh7 and SNU-182 cell proliferation (Figures 3(c) and 3(d)), whereas PCNAP1 upregulation presented an opposite effect (Figures 3(e) and 3(f)). These suggest that PCNAP1 promotes HCC cell proliferation.

### 3.4. PCNAP1 Acts as a ceRNA to Sponge miR-340-5p, which Has Been Confirmed to Inhibit ATF7 Expression in HCC

**Figure 4:**
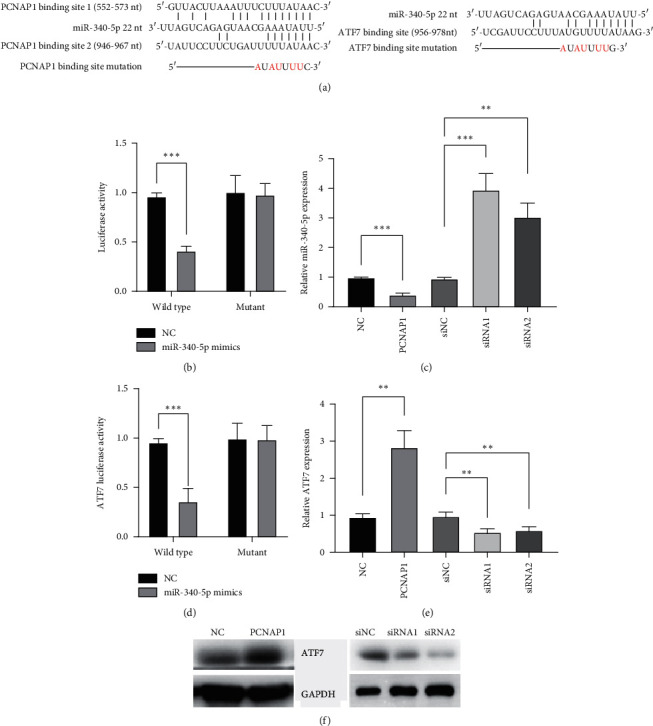
PCNAP1 acts as a ceRNA to sponge miR-340-5p, which has been confirmed to inhibit ATF7 expression in HCC. (a) The binding sites of PCNAP1 and ATF7 to miR-340-5p, respectively, and the mutation of the binding sites. ((b) and (d)) Bars show the luciferase activity in HEK293 cells. Wild type denotes the wild-type binding site. Mutant denotes the mutant binding site. (c) Bars reveal the relative expression of miR-340-5p in SNU-182 cells, detected by qRT-PCR, and U6 served as the internal control. (e) Bars show the relative expression of ATF7 in SNU-182 cells, detected by qRT-PCR, and GAPDH served as the internal control. (f) Western blotting shows the protein expression of ATF7 in SNU-182 cells, and GAPDH served as the internal control. “^∗∗^”, *P* < 0.01; “^∗∗∗^”, *P* < 0.001; “^∗∗∗∗^”, *P* < 0.0001.

Next, we ought to investigate the underlying mechanism by which PCNAP1 promotes HCC cell proliferation. We predicted the candidate miRNAs using miRDB and found 76 miRNAs may target PCNAP1. We also observed that hsa-miR-340-5p had the highest TargetScore. It has been confirmed to be downregulated in HCC and be associated with HBV infection [16], so we considered miR-340-5p being a potential target of PCNAP1 in HCC. The binding site of PCNAP1 to miR-340-5p and the mutant site between them are shown in Figure 4(a). We directly used the luciferase report system to determine whether PCNAP1 directly targets miR-340-5p. The results showed that miR-340-5p mimics significantly inhibited luciferase activity in HEK293 cells which had been transfected with luciferase report vectors containing PCNAP1 fragments of two wild-type binding sites of miR-340-5p, whereas it did not influence luciferase activity in those with two mutant binding sites (Figure 4(b)). Moreover, QRT-PCR experiments revealed that PCNAP1 upregulation significantly inhibited miR-340-5p expression in SUN-182 cells, whereas PCNAP1 knockdown presented an opposite effect (Figure 4(c)). Due to ATF7 being a confirmed target of miR-340-5p in HCC [16], we detected whether PCNAP1 affects ATF7 expression. The results revealed that PCNAP1 upregulation significantly increased the mRNA and protein expression of ATF7, while PCNAP1 knockdown had an opposite effect (Figures 4(d) and 4(e)). These suggest that PCNAP1 regulates the miR-340-5p/ATF7 signaling in HCC.

### 3.5. PCNAP1 Promotes HCC Cell Proliferation via Inhibiting miR-340-5p *In Vitro*

**Figure 5:**
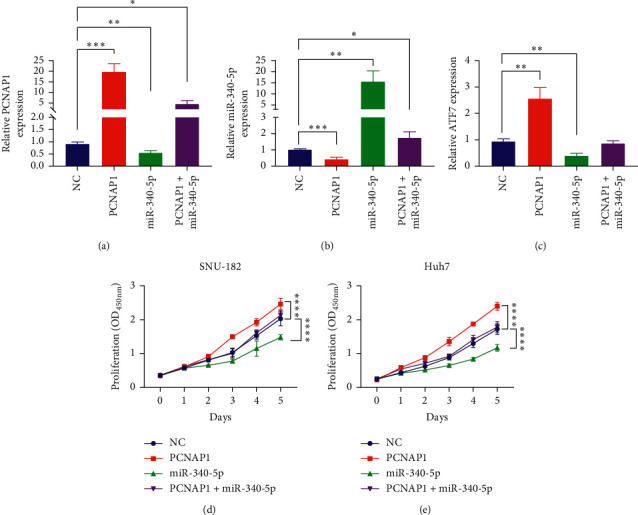
PCNAP1 promotes HCC cell proliferation via inhibiting miR-340-5p *in vitro.* (a-c) Bars show the expression of PCNAP1, miR-340-5p, and ATF7 in SNU-182 cells. GAHPD served as the internal control for PCNAP1 and ATF7, and U6 served as the internal control for miR-340-5p. (d-e) 24 h after transfection, the proliferation of Huh7 and SNU-182 cells at the zeroth to fifth day was measured using a CCK8 reagent. “^*∗*^”, *P* < 0.05; “^∗∗^”, *P* < 0.01; “^∗∗∗^”, *P* < 0.001; “^∗∗∗∗^”, *P* < 0.0001.

Next, we sought to investigate that PCNAP1 promotes HCC cell proliferation via miR-340-5p/ATF7 signaling. PCNAP1-overexpressing vectors and miR-340-5p mimics were cotransfected, as well as separately transfected into SUN-182 cells, and the expression of PCNAP1, miR-340-5p, and ATF7 was detected (Figures 5(a)–5(c)). CCK8 experiments revealed that miR-340-5p mimics significantly reversed PCNAP1-induced proliferation of Huh7 and SNU-182 cells (Figure 5(d)). These suggest that PCNAP1 promotes HCC cell proliferation through regulating miR-340-5p/ATF7 signaling.

### 3.6. The PCNAP1/miR-154/PCNA Signaling Associates with HCC Patient Survival

**Figure 6:**
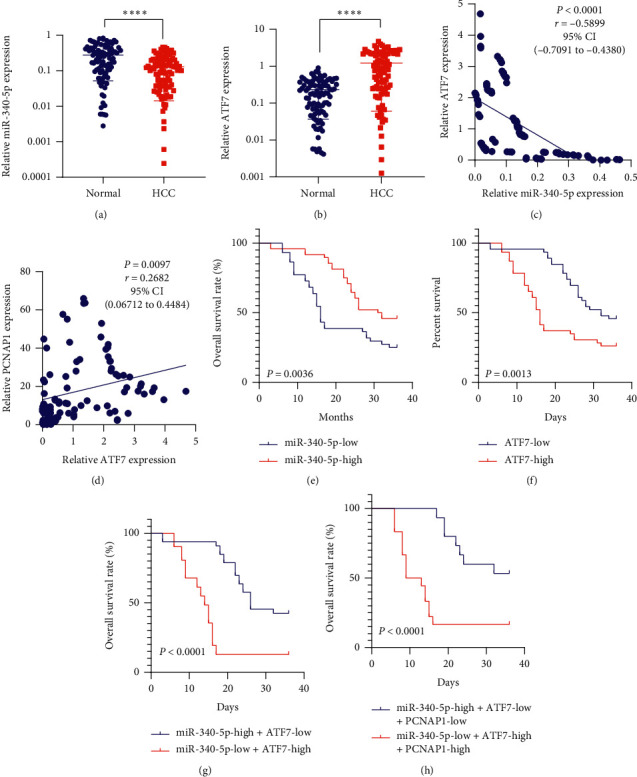
The PCNAP1/miR-154/PCNA signaling associates with HCC patient survival. (a and b) Scatters show relative expression of miR-340-5p and ATF7 mRNA in 92 pairs of HCC and matched normal tissues, detected by qRT-PCR and normalized to GAPDH. (c and d) Scatters and liners show the correlation between the expression of ATF7 mRNA and miR-340-5p and between the expression of PCNAP1 and ATF7 mRNA in 92 HCC tissues. (e and f) 41 HCC patients with low levels of miR-340-5p or high levels of ATF7 mRNA had poorer survival rates. (g) 33 HCC patients with high levels of miR-340-5p and low levels of ATF7 mRNA, and 31 HCC patients with low levels of miR-340-5p and high levels of ATF7 mRNA. (h) 15 HCC patients with high levels of miR-340-5p, low levels of ATF7 mRNA, and low levels of PCNAP1, and 18 HCC patients with low levels of miR-340-5p, high levels of ATF7 mRNA, and high levels of PCNAP1.“^∗∗∗∗^”, *P* < 0.0001.

To evaluate the effect of the PCNAP1/miR-154/PCNA signaling on the survival rates of HCC patients, we analyzed the expression of miR-340-5p and ATF7 mRNA in 92 HCC tissues. MiR-340-5p expression was decreased, but ATF7 mRNA expression was increased in HCC tissues compared with matched normal tissues (Figures 6(a) and 6(b)). Moreover, a negative correlation between ATF7 mRNA expression and miR-340-5p expression and a positive correlation of PCNAP1 expression with ATF7 expression were observed (Figures 6(c) and 6(d)). Given the correlation of the three molecules in HCC, we further analyzed if the combination of the three's expression associates with HCC patient survival. The median expression of each molecule in HCC tissues was used to define their high or low expression, respectively. We found that HCC patients with low expression of miR-340-5p or high expression of ATF7 had poorer survival rates than those with high miR-340-5p expression or low ATF7 expression, respectively (Figures 6(e) and 6(f)). HCC patients with low expression of miR-340-5p and high expression of ATF7 mRNA had poorer survival rates than those with the opposite expression of the two molecules (Figure 6(g)). Moreover, HCC patients with high expression of PCNAP1 and ATF7 and low miR-340-5p expression had poorer survival rates than those with the opposite expression of the three molecules (Figure 6(h)). These suggest that the PCNAP1/miR-154/PCNA signaling associates with the poor survival rates of HCC patients.

## 4. Discussion

Current therapeutic strategies provide a good outcome for patients with early HCC but little benefit for those with advanced HCC [2, 3]. There are a lot of reasons for advanced HCC, such as lack of effective therapeutic targets and drug resistance [6]. Therefore, exploring novel diagnostic biomarkers and therapeutic targets for HCC is important.

In the present study, we demonstrate that lncRNA PCNAP1 is upregulated in HBV-infectious HCC tissues compared with matched normal tissues. These results are consistent with a previous study [15], which showed that PCNAP1 is increased in HBV-infectious HCC versus peritumor. However, whether PCNAP1 expression is also increased in noninfectious HCC remains unclear. Our findings indicate that PCNAP1 expression is increased in noninfectious HCC tissues versus matched normal tissue. In addition, we also found high PCNAP1 expression in HBV-infectious HCC compared with noninfectious HCC, suggesting that PCNAP1 expression is indeed associated with HBV-infectious HCC. However, unexpectedly, PCNAP1 expression between HBV-infectious normal tissue and noninfectious normal tissue has no significant difference. This may be caused by nonsymmetrical infection in the matched normal tissue, but we also observed an increased expression trend in HBV-infectious normal tissues.

Understanding whether PCNAP1 expression signature is associated with HCC patients' survival is important because this signature may be used to predict the outcome of HCC patients. Our results show that high PCNAP1 expression is significantly correlated with poor overall survival of HCC patients, suggesting that PCNAP1 expression signature may be a potential biomarker for the prognosis of HCC patients. An important thing is how to define the high and low expression of PCNAP1 in HCC tissue. ROC cure is well known to assess the detective power of a biomarker [17, 18]. Thus, we got three cut-off values using ROC cure analysis from three different groups of samples. Our findings reveal that using the cut-off values of (1) and (2) to define the high and low expression of PCNAP1 reveals that HCC patients with high expression of PCNAP1 have a poor overall survival rate. This method is also adopted to define the high and low expression of lncRNAs in the circulation [19, 20].

A previous study has demonstrated that PCNAP1 promotes HCC cell proliferation through regulating the miR-154-PCNA axis, which is involved in HBV replication in HCC cells [15]. Although the PCNAP1/miR-154/PCNA signaling has been shown to play important roles in HCC cell proliferation, miR-154 may not be the only target of PCNAP1, because one miRNA can target multiple RNAs; meanwhile, multiple miRNAs can bind to the same RNA. In the present study, we found 76 candidate miRNAs that may be potential targets of PCNAP1 using miRDB, but we consider miR-340-5p being the most potential candidate due to the fact that (1) miR-340-5p ranks the top in TargetScore and (2) miR-340-5p has been shown to be downregulated in HCC and inhibits HCC cell proliferation [16]. Our results confirm that PCNAP1 directly targets miR-340-5p and results in an increased expression of ATF7, consistent with a previous study that miR-340-5p inhibits HCC cell proliferation by directly targeting ATF7 [16]. MiR-340-5p is well known to act as a tumor suppressor in a variety of cancers [16, 21–24]. One of the targets of miR-340-5p in HCC is ATF7, which is involved in cancer cell proliferation [16, 25]. Interestingly, miR-340-5p is downregulated by HBV infection [16]. However, the underlying mechanisms by which HBV infection decreases miR-340-5p expression and whether this is involved in PCNAP1 need to be explored in the future. Our findings showed that HCC patients with high levels of PCNAP1 and ATF7 and low levels of miR-340-5p had poorer survival rates. Thus, our results suggest that the PCNAP1/miR-340-5p/ATF7 signaling not only is involved in HCC cell proliferation but also has a potential for the prognosis of HCC patients.

In conclusion, we highlight that the PCNAP1/miR-340-5p/ATF7 signaling associates with the poor survival rates of HCC patients and plays important roles in HCC cell proliferation, suggesting that the PCNAP1/miR-340-5p/ATF7 signaling may be a potential biomarker for the prognosis of and a therapeutic target for HBV-infectious HCC.

## Figures and Tables

**Table 1:** Clinical characteristics of 92 patients with HCC.

Variable	HBV infection		*P* value
	Yes (*n* = 51)	No (*n* = 41)	
Gender			0.536
Male	28	22	
Female	23	19	
Age			0.391
≥58	26	23	
<58	25	18	
PCNAP1 expression			0.555
High	27	34	
Low	14	17	

Differences among variables were analyzed using the *χ*^2^ test.

**Table 2:** Sequences of oligonucleotides used in this study.

Gene	Type	Sequence (5′-3′)
PCNAP1	F-primer	CACTCCACTCTCTCTTCC
	R-primer	CAGAAAACCGCATCTACC
GAPDH	F-primer	AACGGATTTGGTCGTATTG
	R-primer	GGAAGATGGTGATGGGATT
miR-340-5p	RT-primer	CTCAACTGGTGTCGTGGAGTCGGCAATT
		CAGTTGAG
		AATCAGTC
	F-primer	ACACTCCAGCTGGGTTATAAAGCAATGA
		GA
	R-primer	TGGTGTCGTGGAGTCG
U6	RT-primer	AACGCTTCACGAATTTGCGT
	F-primer	CTCGCTTCGGCAGCACA
	R-primer	AACGCTTCACGAATTTGCGT
ATF7	F-primer	AAGTATCCGTTCCGCCAAGG
	R-primer	GCATTGCACACAAACGGTCT
PCNAP1	siRNA	CCUAGAGAGAUAUCUGCUU
miR-340-5p	Mimics	UUAUAAAGCAAUGAGACUGAUU
miR-340-5p	Inhibitors	AAUCAGUCUCAUUGCUUUAUAA

## Data Availability

The underlying data supporting the results can be obtained from the corresponding author.
